# Two-color imaging of microRNA with enzyme-free signal amplification *via* hybridization chain reactions in living cells†
†Electronic supplementary information (ESI) available: Synthesis and characterization of the nanoprobe, kinetics of the nanoprobe, nuclease stability and MTT assay, and all experimental details. See DOI: 10.1039/c5sc03909f


**DOI:** 10.1039/c5sc03909f

**Published:** 2015-12-07

**Authors:** Lu Li, Jie Feng, Haiyun Liu, Qingling Li, Lili Tong, Bo Tang

**Affiliations:** a College of Chemistry , Chemical Engineering and Materials Science , Collaborative Innovation Center of Functionalized Probes for Chemical Imaging in Universities of Shandong , Key Laboratory of Molecular and Nano Probes , Ministry of Education , Shandong Normal University , Jinan , 250014 , P. R. China . Email: tangb@sdnu.edu.cn ; Fax: +86-531-8618-0017

## Abstract

*In situ* imaging of miRNA in living cells could facilitate the monitoring of the dynamic expression and distribution of miRNA and research on miRNA-related cellular processes and diseases. Given the low expression levels and even down-regulation of cellular miRNA that is associated with some diseases, amplification strategies are imperative for intracellular miRNA imaging. The present paper proposes a non-destructive amplification strategy for use in living cells. This amplification strategy utilizes the enzyme-free hybridization chain reaction (HCR) with graphene oxide (GO) as a carrier to image cellular miRNA. The resulting signal amplification provides excellent recognition and signal enhancement of specific miRNAs in living cells. As the fluorescence quencher and probe carrier, GO enables activation of the signal switch and effective intracellular delivery of amplification reagents. This new imaging method realizes simple, sensitive and non-destructive signal amplification of miRNA in living cells and has an ability to simultaneously image two types of miRNA in the same cell. This method supplies accurate information regarding cellular miRNA-related biological events and provides a new tool for highly sensitive and simultaneous imaging of multiple low-level biomarkers, thereby improving the accuracy of early disease diagnosis.

## Abstract 2

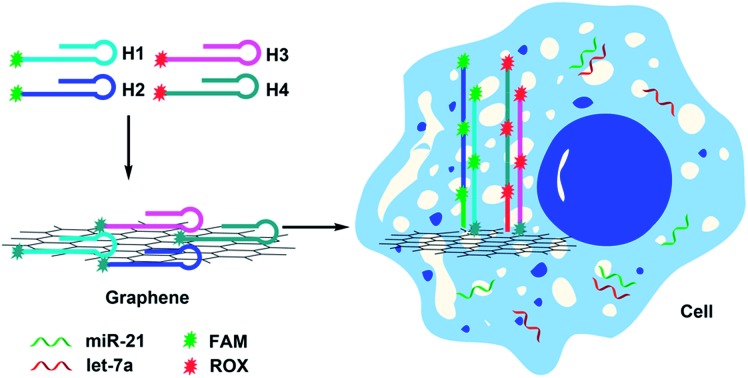
Here, a non-destructive amplification strategy is developed to image miRNAs in living cells, utilizing the enzyme-free hybridization chain reaction (HCR) with graphene oxide (GO) as a carrier. This provides a new tool for highly sensitive and simultaneous imaging of multiple low-level biomarkers, improving the accuracy of early disease diagnosis.

## Introduction

MicroRNAs (miRNAs) are a group of endogenous noncoding RNAs that can regulate the expression of target genes and play significant roles in a diverse range of cellular processes.^1^–^5^ Notably, most miRNAs are expressed in cells at low levels and are vulnerable to enzymatic digestion. Moreover, cellular expression of some low-level miRNAs is even down-regulated when associated with serious diseases.^6^–^8^ Thus, highly sensitive detection of miRNAs is crucial to better understand their roles in cells and to further validate their function in clinical diagnoses. Currently, the real time polymerase chain reaction, northern blot, microarray technology and various new strategies have been developed to improve the sensitivity of miRNAs detection.^9^–^22^ However, most of these methods are only suitable for evaluating homogeneous solutions. Moreover, these methods can not monitor the dynamic expression and distribution of miRNAs in living cells. *In situ* fluorescence imaging technology provides an effective tool for visualizing intracellular miRNA. There is substantial scientific interest in imaging cellular miRNA,^23^–^26^ but only Li *et al.* have reported an amplification strategy based on toehold-initiated rolling circle amplification (RCA) to detect individual miRNAs in single cells.^27^ However, RCA in cells requires the cellular delivery of enzymes and other proteins, such as phi29 DNA polymerase and BSA. Cells may be damaged and even killed by the required fixation and pretreatment, thus presenting a significant challenge for research regarding miRNA-related biological processes in living cells. Effective signal amplification of miRNAs in living cells remains an unresolved challenge.

Thus, it is imperative to design an appropriate signal amplification strategy and choose appropriate bio-friendly carriers to obtain efficient intracellular delivery. Herein, we first introduce an enzyme-free amplification strategy to image low-level miRNA in living cells using the hybridization chain reaction (HCR) with graphene oxide (GO) as the carrier. Because the enzyme is eliminated and its two-stranded products are stable, HCR is an ideal strategy for amplifying and thus detecting various biomolecules.^28^–^31^ Additionally, GO is a good cellular transporter and fluorescence quencher.^32^–^35^ Moreover, HCR products can accumulate on GO, thus enriching the fluorescence signal.^30^ This new imaging method provides simple, sensitive and non-destructive signal amplification of miRNA in living cells and is capable of simultaneously imaging two types of miRNA in the same cell. To our knowledge, this work is the first to amplify the signal of multiple miRNAs in living cells.

## Results and discussion

**Scheme 1:**
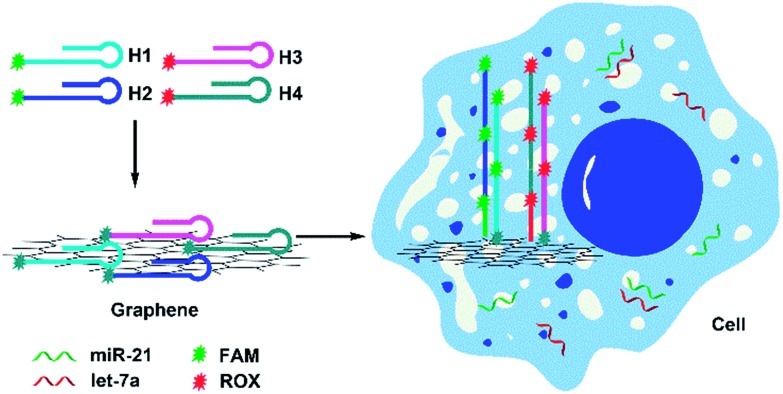
Scheme for amplification and two-color imaging of miRNAs in living cells based on HCR and GO.

The imaging strategy is illustrated in Scheme 1. First, we designed two pairs of hairpin probes labeled with two different fluorescent dyes to image the miRNAs miR-21 and let-7a. The hairpin probes H1 and H2, which each had the fluorophore FAM at the sticky end, were specifically designed according to the miR-21 sequence. The hairpin probes H3 and H4, which were each attached to the fluorophore ROX, were specifically designed according to the let-7a sequence. As a result of noncovalent π–π stacking interactions between the hexagonal cells of graphene and the ring of nucleobases, hairpin DNA probes are closely adsorbed onto the GO surface, forming a probe/GO complex.^36^–^40^ The fluorescence of the dye labeling on the probes was efficiently quenched by GO. Then, the GO, as carrier, transported the hairpin probes into cells *via* non-destructive clathrin-mediated endocytosis.^41^ The hairpin probe/GO complex was used as a fluorescence-sensing platform for imaging miRNAs in living cells. When miR-21 miRNAs were present in cells, each miR-21 molecule could specifically trigger an HCR between H1 and H2, yielding long dsDNA with collected fluorophores. The specific reaction has been described in a previous report.^42^ As a result of weak interaction between the dsDNA and GO, remarkable fluorescence enhancement was obtained. One miR-21 molecule could be effectively amplified by several FAM fluorophores, and green fluorescence was imaged in the cells. In the same manner, let-7a could specifically trigger an HCR between H3 and H4, so that cellular let-7a miRNAs were amplified and imaged by the red ROX fluorescence.

The quenching ability of GO was evaluated using the fluorescence spectra of the dye-labeled hairpin probes in the presence of GO. As shown in Fig. S1,† the addition of 25 μg mL^–1^ GO resulted in complete fluorescence quenching of 5 nM FAM-H1, 5 nM FAM-H2, 5 nM ROX-H3 and 5 nM ROX-H4. At this ratio, the fluorescence of the dye labeling the hairpin probes can be effectively quenched by GO within 1 min (Fig. S2†). The effect of the amount of the hairpin probes on signal intensity and fluorescent background was also investigated simultaneously to obtain an optimal signal-to-noise ratio. The result is shown in Fig. S3.† When the concentration of GO was 25 μg mL^–1^, both the fluorescence signal intensity (red curve) and the fluorescent background (black curve) of the mixture solution increased with increasing concentration of hairpin probes from 1 nM to 10 nM. The inset of Fig. S3† shows the relative fluorescent intensity which is defined as the difference between the fluorescence signal intensity and the fluorescent background. The relative fluorescent intensity increased with increasing concentration of the hairpin probes from 1 nM to 5 nM. Within this concentration range, more hairpin probes may produce stronger signal amplification. At the same time, the fluorescent signal of FAM on H1/H2 could be effectively quenched. When the concentration of H1/H2 was more than 5 nM, the relative fluorescent intensity decreased with increasing H1/H2 concentration, which can be explained by the fact that when hairpin probes were added above 5 nM concentration, GO did not totally quench the fluorescent signal of FAM on H1/H2. Finally, 5 nM hairpin probes and 25 μg mL^–1^ GO were used throughout the entire detection process.

**Fig. 1:**
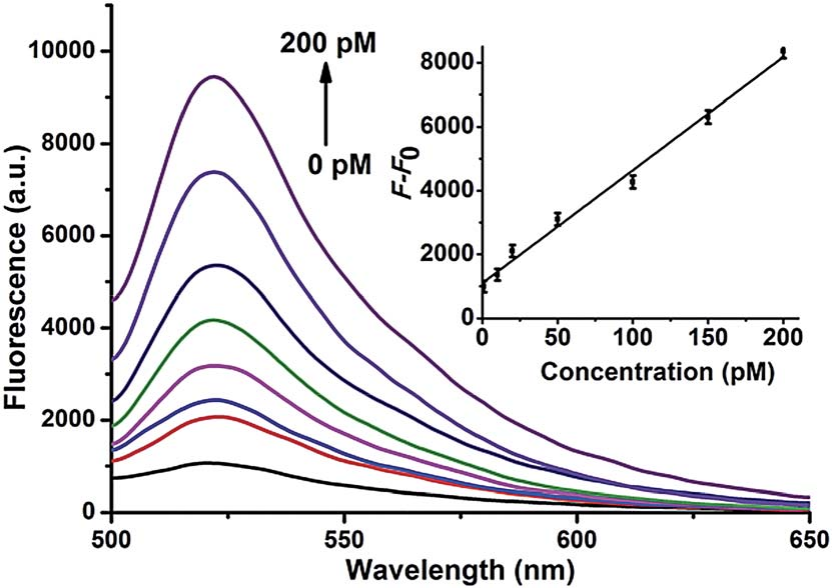
Fluorescence spectra of the mixture solutions with various concentrations of miR-21 measured with 494 nm excitation wavelength. Inset: calibration curves for the fluorescence intensity *versus* corresponding target concentrations (1, 10, 20, 50, 100, 150, 200 pM). Error bars were estimated from three replicate measurements.

The miRNA signal amplification obtained using HCR was verified by fluorescence spectral analysis using the miR-21 as the model target (Fig. S4†). When H1 and H2 were adsorbed onto the GO surface, a faint signal arose from FAM due to the effective fluorescence quenching by GO. The fluorescence was slightly enhanced when only H1 was used as a hairpin probe because of one-step hybridization between miR-21 and H1; however, the strongest fluorescence was obtained after the miRNA reacted with both H1 and H2, indicating that the fluorescence signal indeed arises from miRNA-triggered HCR. Gel electrophoresis was also performed to verify the formation of HCR-generated long dsDNA (Fig. S5†). The ability of this approach for quantitative detection of miRNA *in vitro* was investigated and the result is shown in Fig. 1. The result shows that fluorescent intensity of the mixture solutions increases with increasing concentration of the miRNA miR-21 from 0 to 200 pM. A good linearity was obtained from 0 pM to 200 pM of miRNA. The detection limit of the method was calculated to be 0.18 pM for miRNA on the basis of 3*σ* per slope, which is about five times less than for direct detection without an amplification strategy.^26^

**Fig. 2:**
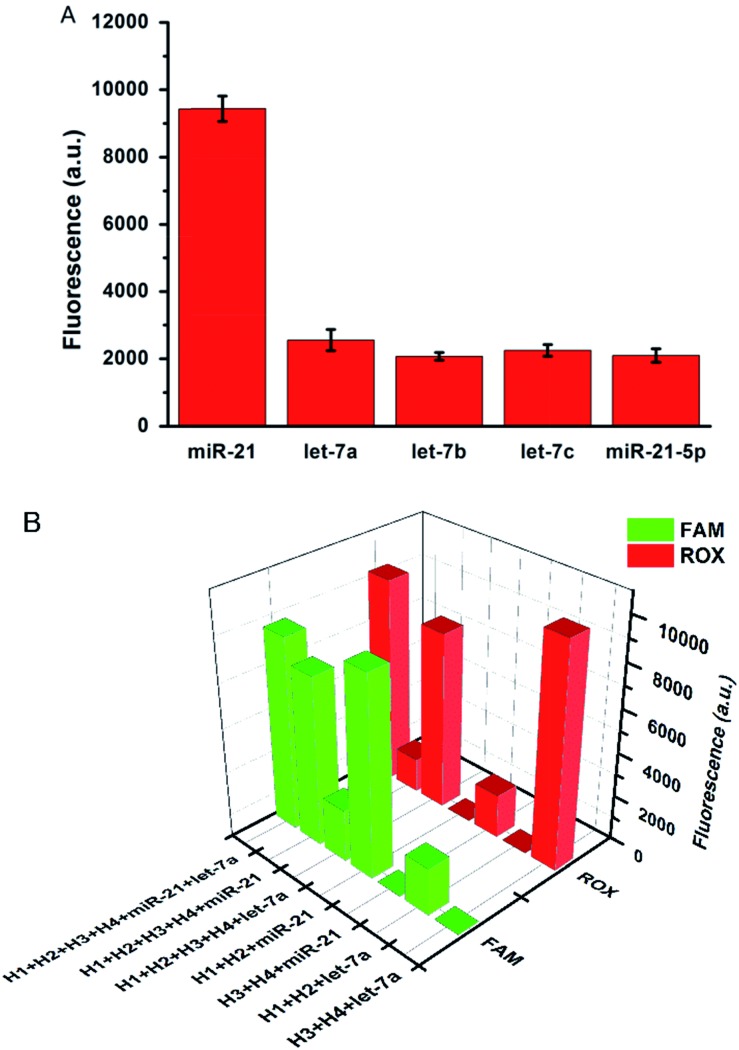
(A) Fluorescence intensities produced by miR-21, let-7a, let-7b, let-7c, and anti-miR-21 with the hairpin probes H1 and H2 specifically designed according to the sequence of miR-21. (B) Fluorescence intensities produced by miR-21 and let-7a with different pairs of hairpin probes.

The specificity of the proposed strategy was also investigated in solution using the following miRNAs at the same concentration: miR-21, let-7a, let-7b, let-7c and anti-miR-21. For the H1 and H2 hairpin probes specifically designed based on the miR-21 sequence, the fluorescence intensity produced by miR-21 was approximately 4-fold of that produced by other miRNAs (Fig. 2A). This result suggests that the proposed miRNA detection approach provides high sequence specificity and demonstrates potential with regard to application in complex biosystems. To ensure that the two pairs of probes do not interfere with each other during two-color imaging, fluorescence spectra were measured after miR-21 and let-7a were incubated with the probes. As shown in Fig. 2B, the green fluorescence of FAM arose solely from the mixture containing miR-21 and H1-H2/GO, whereas the red fluorescence only emanated from the mixture containing let-7a and H3-H4/GO. There was no notable fluorescence enhancement when miRNA was incubated with noncomplementary probes as a control. The DNA probes exhibited excellent specificity for their corresponding target miRNAs, and the fluorescence signals from the HCR products generated from miR-21 and let-7a were not influenced by each other. This method allows multiple miRNAs to be monitored simultaneously with no notable cross-reactivity between targets and noncomplementary probes.

Choosing a bio-friendly carrier capable of efficient intracellular delivery is imperative for *in situ* imaging of miRNAs in living cells. GO is expected to have acceptable biocompatibility and low toxicity. MTT assays were performed to evaluate the cytotoxicity of GO complexes on MCF-7 and MCF-10A cells. The cells were incubated with 0 to 200 μg mL^–1^ GO for 24 h. Fig. S6A† shows that treatment with GO concentrations ≤50 μg mL^–1^ induced little reduction in cell viability. MCF-7 and MCF-10A cells were also incubated with 25 μg mL^–1^ GO for 2–24 h. Cell viability was negligibly affected by GO within 24 h (Fig. S6B†). Thus, to monitor cellular miRNA in the present study, we incubated cells with GO concentrations less than 25 μg mL^–1^ for 8 h, thereby ensuring high cell viability.

**Fig. 3:**
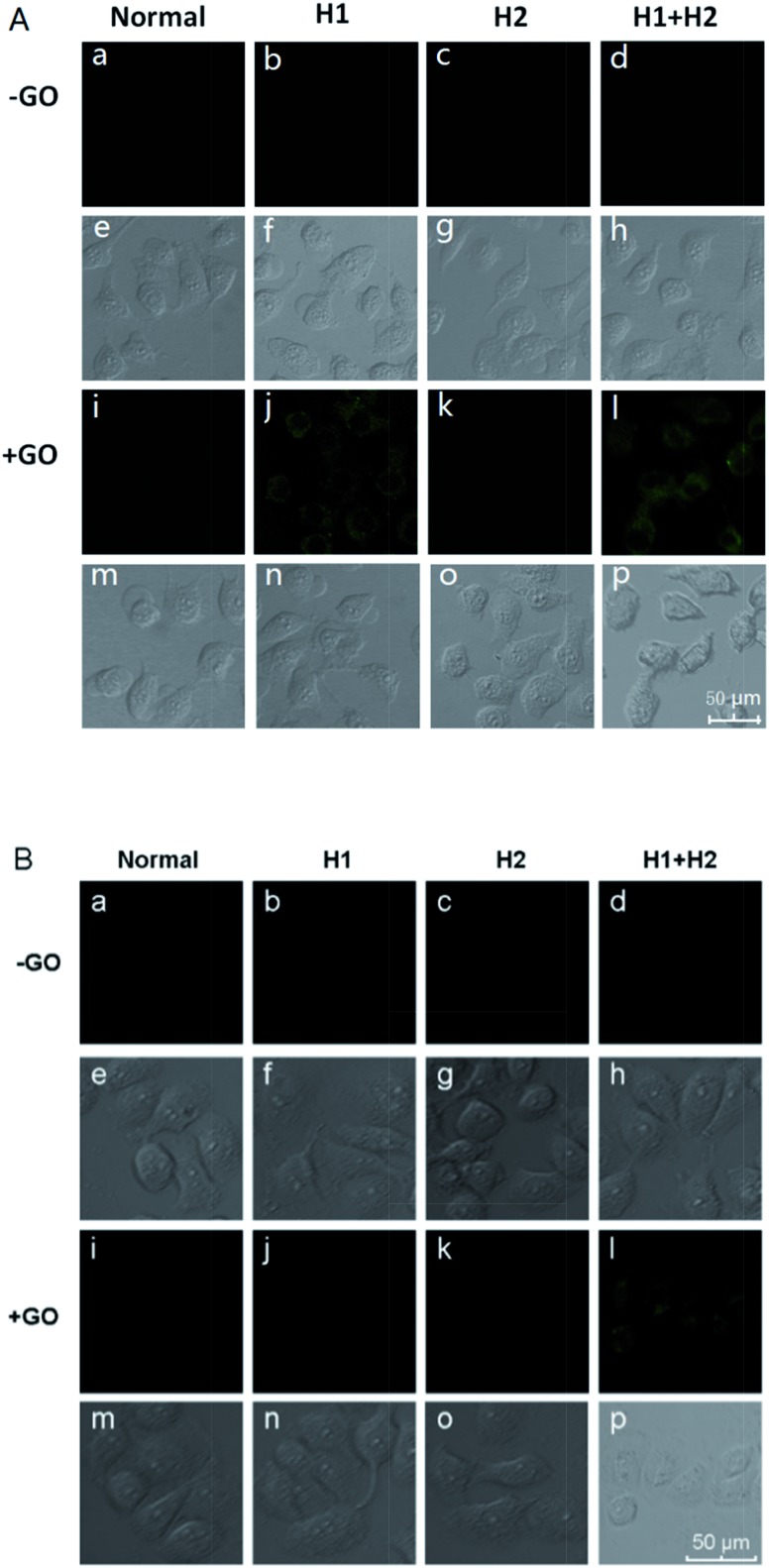
Fluorescence images of miR-21 in MCF-7 cells (A) and MCF-10A cells (B) incubated with different hairpin probes in the presence and absence of GO.

To confirm the amplification and imaging of intracellular miRNAs, MCF-7, which expresses the miRNA miR-21, was separately incubated with H1/GO, H2/GO, and H1 + H2/GO for 8 h. As shown in Fig. 3A, a fluorescence signal derived from the reaction between miR-21 and H1 + H2/GO was clearly observed (Fig. 3A, l), whereas faint fluorescence was observed after the cells were incubated with H1/GO (Fig. 3A, j), and no fluorescence was observed after the cells were incubated with H2/GO (Fig. 3A, k). These results indicate that the hairpin probe was successfully transported intracellularly and that HCR was completed in the living cells, leading to a higher fluorescence signal (Fig. 3A, l) than that obtained *via* the one-step reaction between miRNA and H1/GO (Fig. 3A, j). As controls, MCF-7 cells were also incubated with H1, H2, and H1 + H2 without GO. In these cases, no fluorescence signals were observed (Fig. 3A, b–d), demonstrating that GO is an efficient carrier for transporting probes through cell membranes. GO reportedly enters cells mainly *via* energy-dependent, clathrin-mediated endocytosis.^41^ The above amplification strategy was also applied in MCF-10 cells exhibiting lower expression levels of miR-21. A similar phenomenon was observed (Fig. 3B) except that no fluorescence rather than faint fluorescence was observed after the cells were incubated with H1/GO (Fig. 3B, j). These results demonstrate that it is difficult to image low-level miRNA using a direct probe and target label with a 1 : 1 stoichiometric ratio, but the amplification strategy is advantageous for visualizing low levels of miRNA in living cells. Fluorescence spectral analysis of a large number of MCF-7 and MCF-10A cells incubated with H1 or with H1 + H2 further confirmed that this strategy provided intracellular amplification of miRNAs (Fig. S7†).

**Fig. 4:**
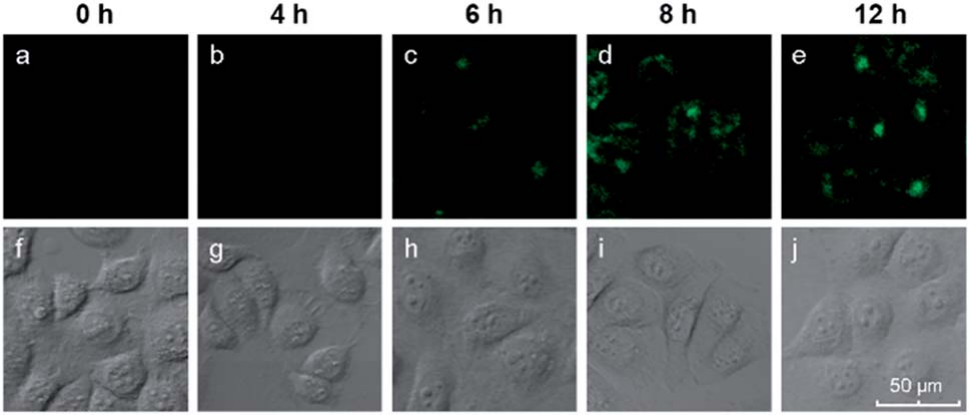
Fluorescence images of miR-21 in MCF-7 cells after different incubation times.

We also investigated the effects of varying the duration of the incubation of the cells with the hairpin probes. MCF-7 cells were incubated with the H1 + H2/GO complex for different time periods from 0 to 12 h, after which images of the cells were obtained with a confocal microscope. The results are shown in Fig. 4. No obvious fluorescence was observed from the cells that were incubated for 4 h (Fig. 4b). When the incubation time was increased to 6 h, fluorescence was observed in the MCF-7 cells incubated with hairpin probes/GO (Fig. 4c). At 8 h, the fluorescence of MCF-7 cells was brilliant (Fig. 4d), and the fluorescence intensities did not increase when the incubation times were increased further (Fig. 4e). These results indicated that the transport of probes by GO and HCR could be completed within 8 h. At 8 h, the cells maintained good activities as indicated by DIC images (Fig. 4i) and MTT assays.

**Fig. 5:**
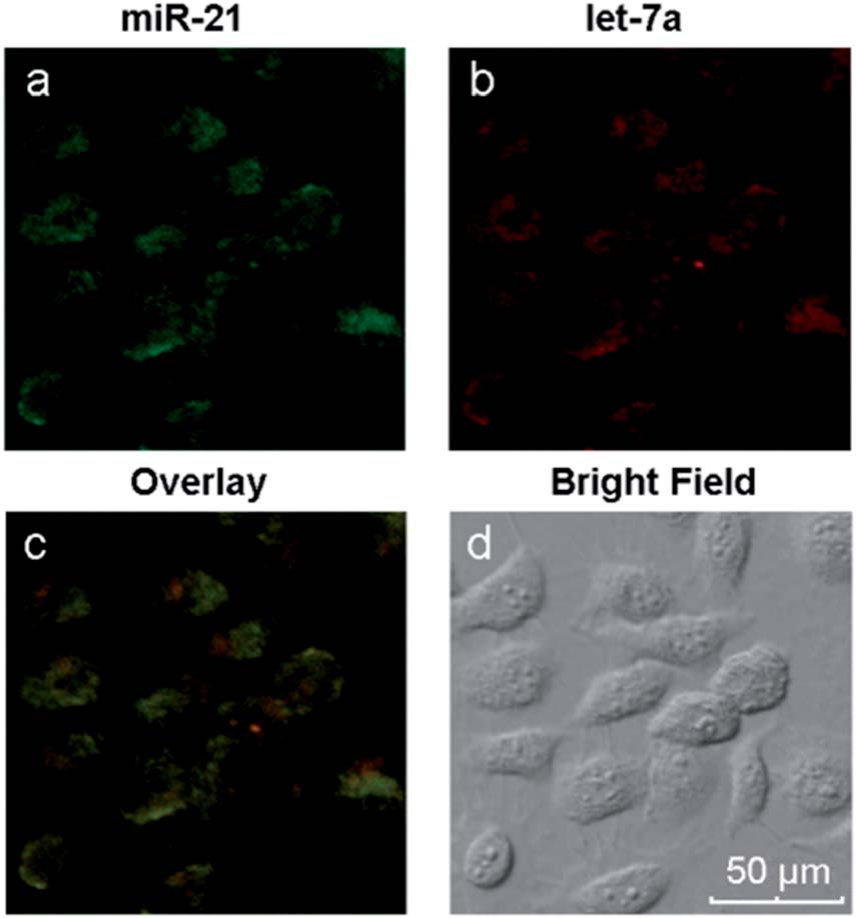
Two-color fluorescence images of miR-21 and let-7a in MCF-7 cells.

Different miRNAs usually regulate important cellular bio-processes collaboratively and simultaneously, and some diseases are associated with multiple miRNA biomarkers. Therefore, simultaneous monitoring of multiple cellular miRNAs is important for the progress of bioresearch and clinical diagnosis. However, due to the low expression levels of some miRNAs, it is difficult to simultaneously detect multiple miRNAs within the same living cell. The biomarkers miR-21, which is highly expressed, and let-7a, which exhibits low expression levels, coexist in MCF-7 cells.^43^–^45^ We have demonstrated the simultaneous signal amplification of miR-21 and let-7a in homogeneous solutions with good specificity. We next verified the appropriateness of the amplification strategy for simultaneously imaging the two miRNAs at different expression levels in the same living cells. First, two pairs of probes (H1 + H2 and H3 + H4) were adsorbed onto the GO surface, forming a probe/GO complex. Then, the MCF-7 cells were incubated with this complex for 8 h and imaged with a confocal microscope. The results are depicted in Fig. 5. The miRNAs miR-21 and let-7a are represented by green and red fluorescence, respectively, in the same cells. The two-color fluorescence images exhibit different amounts and spatial distributions of miR-21 and let-7a in living cells, demonstrating the excellent capabilities of this amplification strategy for imaging multiple intracellular miRNAs. A comparison of the present technique with current imaging methods is shown in Table S2.† The images also demonstrate both the potential to utilize this amplification strategy for early cancer detection and the resulting possible improvements in diagnostic accuracy and thus avoidance of false positive results.

## Conclusions

In conclusion, the present study reports a new method for amplifying and visualizing low levels of miRNA in living cells for the first time. The assay is technologically easy to implement in living cells because the amplification method is enzyme free and thermal cycling is not needed. When used as a carrier, GO can transport the DNA probes into living cells in a non-destructive manner, thus improving the biological accuracy of the obtained information. Moreover, the amplification method described herein can image multiple tumor-related miRNA biomarkers in living cells, which would avoid false positive results. This method will benefit further studies of miRNA-related bioprocesses and will provide a new tool for highly sensitive and simultaneous imaging of multiple low-level biomarkers, which should improve the accuracy of diagnosing diseases in their early stages.

## Supplementary Material

Supplementary information
