# Supplementary material for: Two-color imaging of microRNA with enzyme-free signal amplification via hybridization chain reactions in living cells
Source: Chem Sci. 2015 Dec 7;7(3):1940–5. doi: 10.1039/c5sc03909f (PMC5966800; doi:10.1039/c5sc03909f)
Supplement: Supplementary file 1 [file SC-007-C5SC03909F-s001.pdf]

Electronic Supplementary Material (ESI) for Chemical Science.
This journal is © The Royal Society of Chemistry 2015

Supporting Information

Two-color Imaging of MicroRNA with Enzyme-Free Signal Amplification via Hybridization Chain Reaction in Living Cells

Lu Li, Jie Feng, Haiyun Liu, Qingling Li, Lili Tong, Bo Tang*

College of Chemistry, Chemical Engineering and Materials Science, Collaborative Innovation Center of Functionalized Probes for Chemical Imaging, Key Laboratory of Molecular and Nano Probes, Ministry of Education, Shandong Normal University, Jinan, 250014 Shandong, People's Republic of China

Experimental Section

Materials and Apparatus. HPLC-purified hairpin probes and microRNAs were purchased from TaKaRa Biotechnology Co., Ltd. (Dalian, China). The sequences of the hairpin probes and microRNAs are listed in Table 1. Diethylpyrocarbonate (DEPC)-treated deionized water, TE buffer and ribonuclease inhibitor were obtained from TaKaRa Biotechnology Co., Ltd. (Dalian, China). Graphene oxide (GO) was purchased from XF Nano, Inc. (Nanjing, China). Fetal bovine serum was purchased from Gibco (Carlsbad, CA, USA). The RPMI 1640 and MirVana miRNA Isolation Kit were purchased from Life Technologies (Carlsbad, CA, USA). DEPC-treated deionized water was used in all experiments.

Table 1 Sequences of Hairpin Probes and MicroRNAs

Name	Sequences (5'-3')
H1	ATCAGACTGATGTTGACAAAGTTCAACATCAGTCTGATAAGCTA-<br>FAM
H2	FAM -<br>ACTTTGTCAACATCAGTCTGATTAGCTTATCAGACTGATGTTGA
H3	AGTAGGTTGTATAGTTCAAAGTAACTATACAACCTACTACCTCA -<br>ROX
H4	ROX -<br>ACTTTGAACTATACAACCTACTTGAGGTAGTAGGTTGTATAGTT
miR-21	UAGCUUAUCAGACUGAUGUUGA

let-7a	UGAGGUAGUAGGUUGUAUAGUU
let-7b	UGAGGUAGUAGGUUGUGUGGUU
let-7c	UGAGGUAGUAGGUUGUAUGGUU
miR-21-5p	UCAACAUCAGUCUGAUAAGCUA

Gel electrophoresis was performed using a DYCZ-24DN Electrophoresis Cell (Liuyi, Beijing, China) and GelDoc-It Imaging Systems (UVP, Cambridge, UK). Fluorescence spectra were obtained using a Cary Eclipse Fluorescence Spectrophotometer (Varian, CA). Absorbance was measured using a microplate reader (RT 6000, Rayto, USA) for the MTT assay. Confocal fluorescence imaging was performed with a TCS SP5 confocal laser scanning microscope (Leica Co., Ltd. Germany) with an objective lens (20×).

Hybridization Chain Reaction. The hybridization chain reaction was performed in 200 μL of reaction mixture containing 5 nM hairpin probe 1 and 5 nM hairpin probe 2, 4 U RNase inhibitor, 1× SPSC buffer [0.75 M NaCl and 50 mM Na_2HPO_4, pH 7.4], and target miRNAs at different concentrations. Before the reaction, each hairpin probe was incubated at 95 °C for 5 min and then slowly cooled to room temperature over 1 h to enable the probe to perfectly fold into a hairpin structure. After annealing, the hairpin probes were added to the mixture and incubated for 4 h at room temperature. Subsequently, GO (25 μg/mL) was added to the system, and the final mixture was incubated for another 30 min.

Fluorescence Spectra. The fluorescence spectra were obtained using a Cary Eclipse Fluorescence Spectrophotometer. Fluorescence of FAM-labeled H1 and H2 was excited at 494 nm, and the resulting spectra were recorded between 500 nm and 650 nm. The fluorescence emission maximum occurred at 521 nm. The fluorescence of ROX-labeled H3 and H4 was excited at 587 nm, and the resulting spectra were recorded between 595 nm and 750 nm. The fluorescence emission maximum occurred at 607 nm.

Gel Electrophoresis Analysis. Amplification products were analyzed by 12% PAGE. The gel was placed in 1× electrophoresis Tris-borate-EDTA (TBE) buffer at 100 V for 1 h and stained with SYBR Green I for 15 min. Gel imaging was performed using a UVP GelDoc-It Imaging System.

Cell Culture. MCF-7 and MCF-10A cells were cultured according to instructions provided by American Type Culture Collection. Cells were grown in Dulbecco′s modified Eagle′s medium (DMEM) (HyClone, 100 U/mL penicillin and 100 μg/mL streptomycin) plus 10% fetal bovine serum (FBS, Gibco) and maintained at 37 °C in a humidified atmosphere of 5% CO_2 and 95% air.

MTT Assay. MTT assays were performed to investigate GO cytotoxicity. MCF-7 cells (1×10^6 cells/well) were dispersed within replicate 96-well microtiter plates to a total volume of 200 μL well−1. Plates were maintained at 37 °C in a 5% CO_2/95% air incubator for 24 h. After the original medium was removed, the MCF-7 cells were incubated with GO (0, 3.125, 6.25, 12.5, 25, 50, 100, or 200 μg/ml) for 8 h. (The cells incubated with only the culture medium served as controls.) The cells were washed with PBS three times, after which 100 μL of MTT solution (0.5 mg/mL in PBS) was added to each well. After 4 h, the remaining MTT solution was removed, and 150 μL of DMSO was added to each well to dissolve the formazan crystals. Subsequent absorbance measurements were obtained at 490 nm using an RT 6000 microplate reader.

Imaging of miRNA in Living Cells. Fluorescence imaging was performed using a Leica TCS SP5 inverted confocal microscope (Leica, Germany). Cellular images were acquired using a 20× objective. The FAM-labeled probe was excited by a 488-nm laser, and the resulting fluorescence was detected from 495 nm to 550 nm. The ROX-labeled probe was excited by a 543-nm laser, and the resulting fluorescence was detected from 580 nm to 750 nm.

Detecting the Fluorescence Intensities of the Cellular Reaction Product. MCF-7 cells and MCF-10A cells were incubated with the probe/GO complex for 8 h. Subsequently, SDS solution (0.1 mol/L) was added to the mixture to achieve an SDS concentration of 20 mmol/L. After the cells were lyzed, the solution was centrifuged (2000 r/min, 5 min) three times, and each supernatant was collected. Fluorescence of the reaction product was excited at 494 nm, and spectra were recorded between 500 nm and 600 nm.

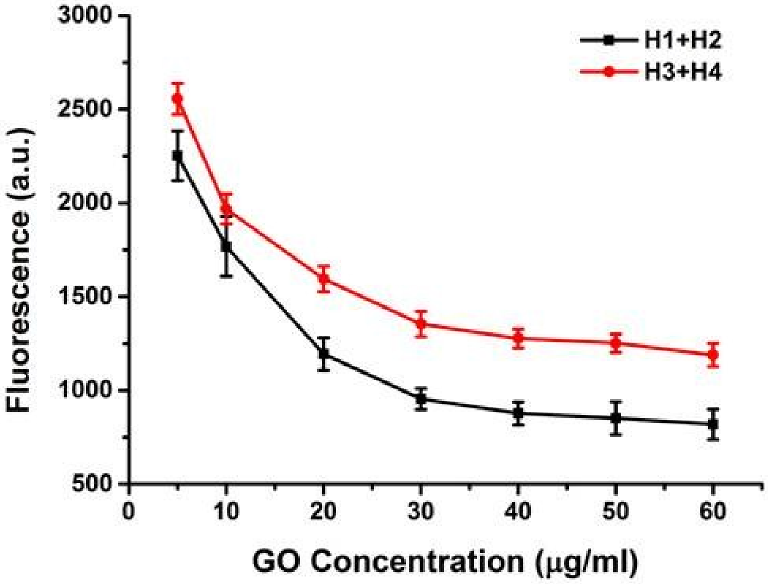

Figure S1. Quenching effect of GO at various concentrations on the fluorescence of H1/H2 and H3/H4. The concentration of H1/H2 and H3/H4 is 5 nM. GO concentrations ranged from 5 μg/mL to 60 μg/mL.

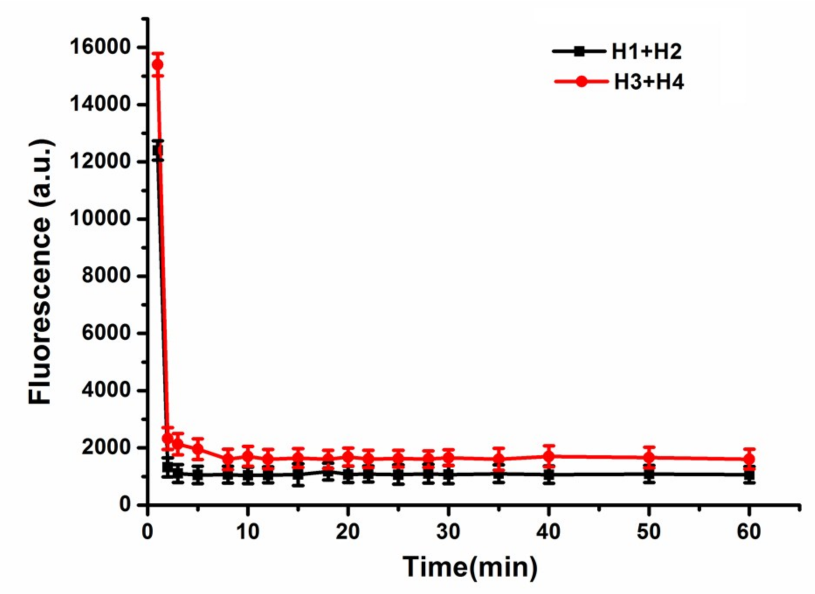

Figure S2. Kinetic characterization of fluorescence quenching. The fluorescence of FAM and ROX at the sticky terminals of the hairpin probes can be effectively quenched by GO within 1 min. The concentrations of H1, H2, H3 and H4 are each 5 nM. The concentration of GO is 25 μg/mL.

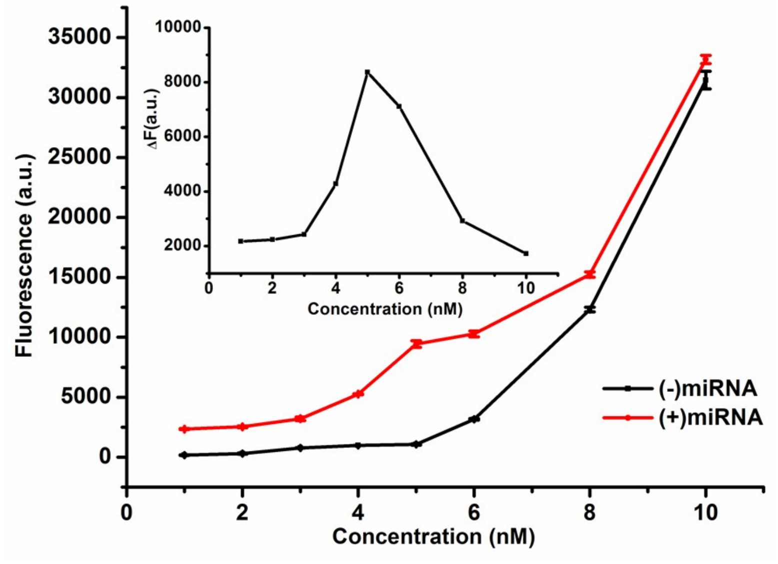

Figure S3. Fluorescence spectra of the reaction product after incubation of miR-21 with different concentration of H1/H2 probes. The concentrations of H1 and H2 are each 1, 2, 3, 4, 5, 6, 8, 10 nM. The concentration of GO is 25 μg/mL. The concentration of miR-21 is 200 pM.

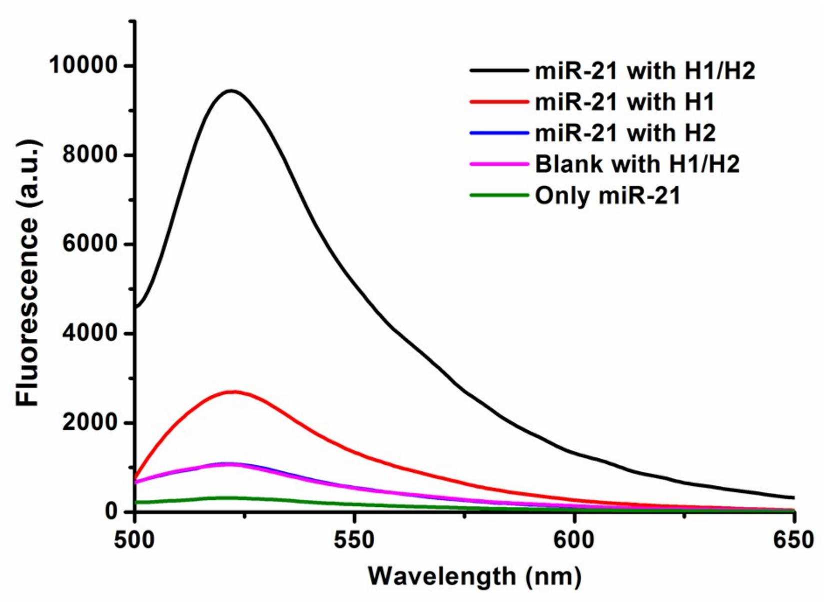

Figure S4. Fluorescence spectra of the reaction product after incubation of miR-21 with different probes. The concentrations of H1 and H2 are each 5 nM. The concentration of GO is 25 μg/mL. The concentration of miR-21 is 200 pM.

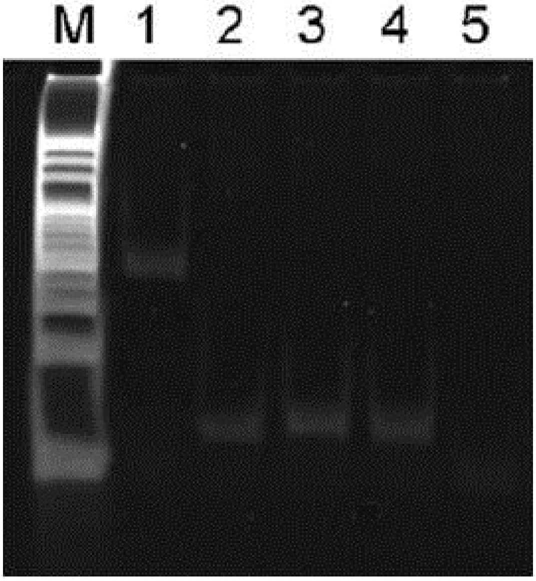

Figure S5. Gel electrophoresis analysis after miR-21 incubation with different probes. Lane 1: 50 nM H1+50 nM H2+5 nM miR-21; lane 2: 50 nM H1+50 nM H2; lane 3: 100 nM H1; lane 4: 100 nM H2; lane 5: 100 nM miR-21. M indicates the marker.

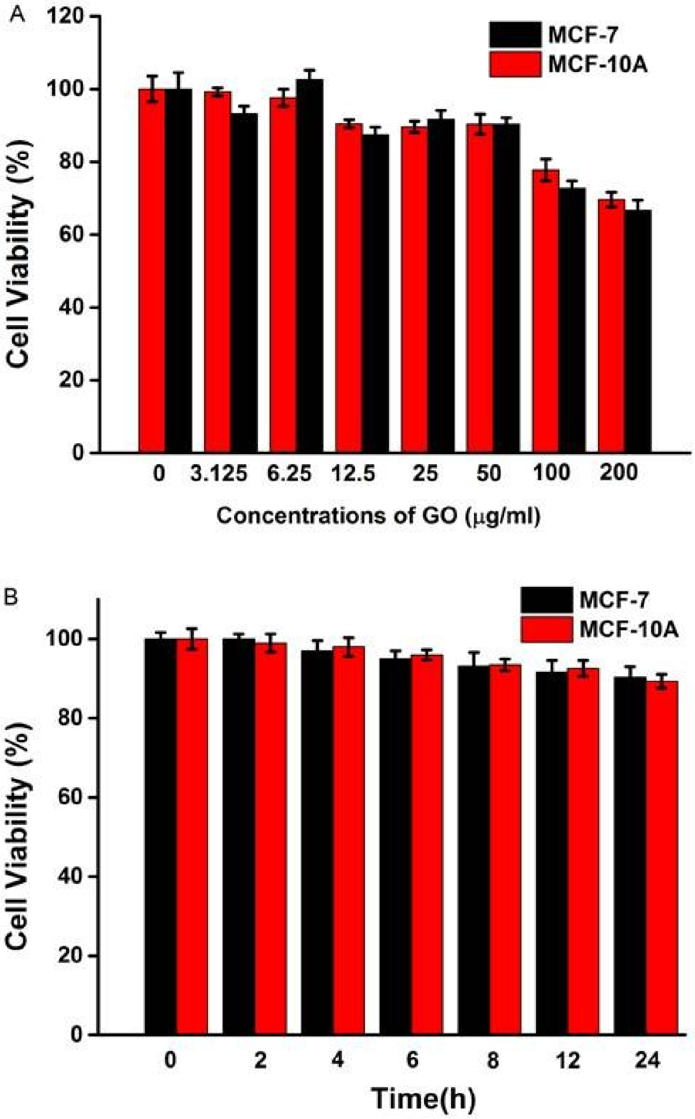

Figure S6. Cytotoxicity induced by GO in MCF-7 and MCF-10A cells.

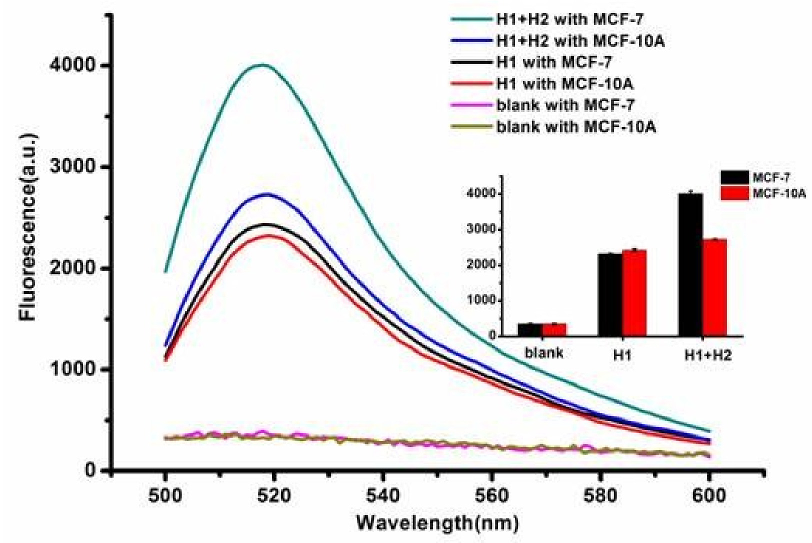

Figure S7. Fluorescence spectra of the reaction product of miR-21 after lysis of MCF-7 and MCF-10A cells.

Table 2 comparison of the present technique with current imaging methods

Imaging Methods	Signal amplification	Multi-components	Living cell	Refs
Dual optical imaging	–	–	+	1
Molecular imaging	–	–	+	2
polyethylenimine-grafted graphene nanoribbon	–	–	+	3
Peptide Nucleic Acid and Nano Graphene Oxide	–	+	+	4
Rolling Circle Amplification	+	–	–	5
This work	+	+	+	

References

1. W. J. Kang, Y. L. Cho, J. R. Chae, J. D. Lee, B. A. Ali, A. A. Al-Khedhairy, C. H. Lee and S. Kim, *Biomaterials*, 2012, **33**, 6430-6437.

2. J. K. Kim, K. J. Choi, M. Lee, M. H. Jo and S. Kim, *Biomaterials*, 2012, **33**, 207-217.

3. H. Dong, L. Ding, F. Yan, H. Ji and H. Ju, *Biomaterials*, 2011, **32**, 3875-3882.

4. S. R. Ryoo, J. Lee, J. Yeo, H. K. Na, Y. K. Kim, H. Jang, J. H. Lee, S. W. Han, Y. Lee, V. N. Kim and D. H. Min, *ACS nano*, 2013, **7**, 5882-5891.

5. R. Deng, L. Tang, Q. Tian, Y. Wang, L. Lin and J. Li, *Angew. Chem., Int. Ed.*, 2014, **53**, 2389-2393.
